# Macular Pigment Response to Lutein, Zeaxanthin, and Meso-zeaxanthin Supplementation in Open-Angle Glaucoma: A Randomized Controlled Trial

**DOI:** 10.1016/j.xops.2021.100039

**Published:** 2021-07-11

**Authors:** James Loughman, Ekaterina Loskutova, John S. Butler, We Fong Siah, Colm O’Brien

**Affiliations:** 1 Centre for Eye Research Ireland, School of Physics, Clinical and Optometric Sciences, Environmental Sustainability and Health Institute, Technological University Dublin, Dublin, Ireland; 2 Centre for Eye Research Ireland, School of Mathematical Sciences, Environmental Sustainability and Health Institute, Technological University Dublin, Dublin, Ireland; 3 Mater Misericordiae University Hospital, Dublin, Ireland

**Keywords:** Glaucoma, Lutein, Macular carotenoids, Macular pigment, Meso-zeaxanthin, MPOD, Zeaxanthin, AMD, age-related macular degeneration, BMI, body mass index, CAREDS, Carotenoids in Age-Related Eye Disease Study, cpd, cycles per degree, CS, contrast sensitivity, CSg, contrast sensitivity under glare, dB, decibel, ENIGMA, European Nutrition in Glaucoma Management, GAL-9, Glaucoma Activities Limitation 9 questionnaire, GCC, ganglion cell complex, HVF, Humphrey visual field, logCS, logarithm of contrast sensitivity units, LZQ, Lutein and Zeaxanthin Questionnaire, MD, mean deviation, MMSE, Mini-Mental State Examination, MP, macular pigment, MPOD, macular pigment optical density, MSE, mean square error, OAG, open-angle glaucoma, RGC, retinal ganglion cell, RNFL, retinal nerve fiber layer, VA, visual acuity, VAR, visual acuity rating

## Abstract

**Purpose:**

To evaluate macular pigment response to carotenoid supplementation in glaucomatous eyes.

**Design:**

Double-masked, randomized, placebo-controlled clinical trial, the European Nutrition in Glaucoma Management Study (ClinicalTrials.gov identifier, NCT04460365).

**Participants:**

Sixty-two participants (38 men, 24 women) with a diagnosis of open-angle glaucoma were enrolled. Forty-two were randomized to receive the active supplement, 20 participants were allocated to placebo.

**Methods:**

Macular pigment optical density (MPOD) was measured by autofluorescence using the Heidelberg Spectralis scanning laser ophthalmoscope. Macular pigment optical density volume within the central 6° of retinal eccentricity as well as MPOD at 0.23°, 0.51°, 0.74°, and 1.02° were recorded at baseline and at 6-month intervals over 18 months. Visual function was assessed using visual acuity, mesopic and photopic contrast sensitivity under glare conditions, photo stress recovery time, microperimetry, and Glaucoma Activities Limitation 9 questionnaire. Advanced glaucoma module scans of retinal nerve fiber layer thickness and ganglion cell complex thickness over the central 6° of retinal eccentricity also were completed at each study visit.

**Main Outcome Measures:**

Change in MPOD after supplementation with 10 mg lutein, 2 mg zeaxanthin, and 10 mg meso-zeaxanthin or placebo over 18 months.

**Results:**

A mixed-model repeated measures analysis of variance revealed a statistically significant increase in MPOD volume (significant time effect: *F*(3,111) = 89.31, mean square error (MSE) = 1656.9; *P* < 0.01). Post hoc *t* tests revealed a significant difference in MPOD volume at each study visit for the treatment group (*P* < 0.01 for all), but no change in the placebo group (*P* > 0.05 for all). A statistically significant increase in mesopic contrast sensitivity under glare conditions was noted at 18 months in the treatment group, but not placebo. No other structural or functional changes were observed. No serious adverse events were noted during the trial.

**Conclusions:**

Macular pigment can be augmented in glaucomatous eyes by supplementation with a formulation containing the carotenoids lutein, zeaxanthin, and meso-zeaxanthin. The greatest relative benefit was observed in those with the lowest baseline levels, but increases were noted across all participants and each retinal eccentricity. The potential benefits of MP augmentation for macular health in glaucoma merit further long-term evaluation.

Glaucoma is a neurodegenerative disease characterized by progressive retinal nerve fiber axon degeneration and retinal ganglion cell (RGC) death.^1^ Although glaucoma traditionally is considered as a disorder affecting peripheral vision, central (or macular) RGC losses are common and can occur early in the disease.^2^ Consequently, the functional losses caused by glaucoma impact various aspects of daily life,^3^ including everyday tasks that require good near and central vision such as reading,^4^ mobility,^5^ and driving.^6^^,^^7^ Furthermore, as a leading cause of vision impairment and blindness,^8^^,^^9^ the health impact associated with vision loss in glaucoma is substantial. Notably, glaucoma affects health-related quality of life^10^^,^^11^ and influences health status more broadly as a contributing factor to falls,^12^ motor vehicle collisions,^13^ mental health disorders,^14^ and cognitive health decline.^15^, ^16^, ^17^, ^18^ The effective control of glaucoma, alleviation of symptoms, and avoidance of vision impairment therefore can deliver important socioeconomic and health impact.

Macular pigment (MP), comprising the carotenoids lutein, zeaxanthin, and meso-zeaxanthin, is highly concentrated at the fovea.^19^ Macular pigment’s constituent carotenoids have been studied extensively for their role in eye health, particularly in age-related macular degeneration (AMD)^20^ and, more recently, in diabetes,^21^^,^^22^ cataract,^23^ and glaucoma.^20^^,^^24^^,^^25^ Investigations into glaucoma have demonstrated that MP optical density (MPOD) is lower in the glaucomatous eye.^24^, ^26^, ^25^ Given the possibility that MP levels may be depleted in glaucoma, the potential benefits of macular carotenoid supplementation for ocular health and visual function in glaucoma merit investigation. The capacity for MP levels to be augmented through carotenoid supplementation in glaucomatous eyes has yet to be established and represents an important precursor to any functional or health-related benefits that may accrue.

The European Nutrition in Glaucoma Management (ENIGMA) trial was designed to evaluate the MP response to supplementation with lutein, zeaxanthin, and meso-zeaxanthin among individuals with open-angle glaucoma (OAG). Additional exploratory analyses of the functional and structural response to supplementation also were prioritized to inform sample size and trial design considerations for future trials to explore the potential long-term neuroprotective impact of macular carotenoid supplementation in glaucoma.

## Methods

### Trial Design

The ENIGMA study (ClinicalTrials.gov identifier, NCT04460365) comprised a randomized, placebo-controlled, double-masked clinical trial designed to establish the MP response to supplementation with lutein, zeaxanthin, and meso-zeaxanthin over an 18-month period. Research ethics committee approval was obtained from the Mater Misericordiae Institutional Review Board and from Technological University Dublin Research Ethics and Integrity Committee. Written informed consent was obtained from all participants, and the study adhered to the tenets of the Declaration of Helsinki. Recruitment was completed at the Mater Misericordiae University Hospital and Mater Private Hospital (Dublin, Ireland), whereas study visits were conducted at the Centre for Eye Research Ireland, a dedicated academic clinical trial center at Technological University Dublin.

### Participants

Trial eligibility criteria included a confirmed diagnosis of OAG (including primary OAG, normal-tension glaucoma, pseudoexfoliative glaucoma, and pigment dispersion glaucoma), age older than 18 years, visual acuity (VA) of less than 0.3 logarithm of the minimum angle of resolution, and ability to give informed consent, to make the required study baseline and follow-up visits, and to adhere to trial protocol. Any volunteer exhibiting signs of underlying ocular disease, such as AMD, diabetic retinopathy, or moderate to significant cataract; a history of any type of dementia or other significant systemic condition that might affect capacity to complete the trial; a history of consumption of a dietary macular pigment supplement (containing lutein, zeaxanthin, or meso-zeaxanthin) in the past 6 months; or the presence of a short-wavelength filtering intraocular lens were excluded. All volunteers also were screened for cognitive impairment using the Mini-Mental State Examination and were excluded if a Mini-Mental State Examination score of less than 27 was recorded.

### Randomization and Intervention

Study participants were assigned randomly to intervention groups using a block randomization (block size, 6; randomization ratio, 2:1 with no stratification) strategy to receive a dietary MP supplement or placebo for 18 months. The randomization sequence was generated by the study statistician (J.S.B.), and a research assistant not otherwise involved in the study performed random allocation to intervention groups based on this randomization sequence at the Centre for Eye Research Ireland. The study investigator (E.L.) received a box of supplements (containing 200 capsules) for each study participant, labelled only with the participant identification number. Allocation concealment was achieved using sequentially numbered prelabelled white plastic drug containers of identical appearance. The randomization sequence was revealed after a masked database review after study completion.

The supplement was provided as a softgel capsule containing 10 mg lutein, 10 mg meso-zeaxanthin, and 2 mg zeaxanthin in a sunflower oil suspension (commercially available as Macushield, provided by Thompson & Capper Ltd, Runcorn, United Kingdom, and prepared by EuroCaps Limited, Tredegar, United Kingdom). This supplement has been deemed safe, with renal, liver, lipid, hematologic, and inflammatory biomarkers all unaffected by supplementation at these concentrations.^27^ The overall concentration is also well below the acceptable daily intake for meso-zeaxanthin, which has been suggested as 3 mg/1 kg body weight per day.^28^ The placebo was provided as a softgel capsule, identical in appearance to the active supplement, but containing sunflower oil only (provided by EuroCaps Limited). One capsule was to be ingested daily with a meal.

### Main Outcome Measures

The primary outcome measure was change in MPOD volume in response to supplementation over 18 months, assessed using fundus autofluorescence. Secondary (exploratory) outcome measures included structural and functional response to supplementation over the study period.

### Trial Procedures

At the baseline study visit, demographic characteristics including age, sex, history of smoking, education years, and clinical characteristics including body mass index and waist-to-hip ratio were recorded for each participant. Dietary intake of lutein, zeaxanthin, and meso-zeaxanthin was assessed at baseline and at the final study visit using the validated LZQ screener.^29^ Vision-related activity limitation associated with glaucoma was assessed at the baseline and final study visit using the Glaucoma Activity Limitation 9 questionnaire, a shortened version of the Glaucoma Quality of Life questionnaire originally developed by Nelson et al.^30^ Macular pigment optical density and functional and structural measurements were completed at the baseline visit and at 6-month intervals thereafter until completion at 18 months. Visual acuity was measured using a computer-generated Early Treatment Diabetic Retinopathy Study chart. The eye with the best VA was chosen as the study eye. Where equal VA was recorded and both eyes were deemed eligible for inclusion, the dominant eye was selected as the study eye.

### Macular Pigment Optical Density Measurement

Macular pigment optical density was measured using a Heidelberg Spectralis HRA + OCT MultiColor device (Heidelberg Engineering GmbH). This technique was selected based on its proven test–retest reliability and reproducibility,^31^ a key requirement for longitudinal evaluation of change in MPOD. This device uses a confocal scanning laser ophthalmoscope and 2 excitation wavelengths (486 nm [blue light, which is well absorbed by MP] and 516 nm [green light, absorption of which by MP is low]). Autofluorescence images of the central 30° area of the retina were recorded for both wavelengths. Macular pigment density maps then were computed by the Heidelberg Eye Explorer software (HEYEX version 1.9.13.0) by analyzing the autofluorescence images obtained at both wavelengths, along with a parafoveal reference point set at 6° retinal eccentricity. Measurements were obtained in a darkened room 30 minutes after pupillary dilation of the study eye (Tropicamide 0.5% w/v; Bausch + Lomb). Macular pigment optical density volume within the central 6° of retinal eccentricity as well as MPOD at 0.23°, 0.51°, 0.74°, and 1.02° retinal eccentricity were obtained from the resulting MPOD plots. In addition, standard deviations of MPOD at each retinal eccentricity were extracted manually using a freely available graph reader (graphreader.com) and used as an indicator of image quality, with a standard deviation of 0.11 set as the cutoff limit for image quality acceptability, as recommended previously.^32^

An exploratory analysis of the visual function and ocular health response to macular carotenoid supplementation was conducted by monitoring visual performance and retinal integrity at each study visit using a range of functional and structural tests.

### Visual Function Measurements

Visual performance was assessed using a range of tests including glare disability, microperimetry, and a quality-of-life questionnaire. Performance under glare conditions was the primary functional area of interest and was assessed using the functional vision analyzer Optec 6500 (Stereo Optical) under photopic (85 cd/m^2^) and mesopic (3.0 cd/m^2^) conditions, as described in detail elsewhere.^33^ Briefly, the test consisted of a series of sine-wave grating charts at 5 spatial frequencies (1.5 cycles per degree [cpd], 3 cpd, 6 cpd, 12 cpd, and 18 cpd) and 9 levels of contrast in 0.15 log contrast sensitivity (CS) decrements. A circumferential internal glare source consisting of 12 white light-emitting diodes (set to an intensity luminance of 42 lux) arranged in a circle around the sine-wave charts was used to create glare disability. To evaluate retinal sensitivity in the macular region, the MAIA microperimetry device was used (CenterVue). A customized examination mode with 19 points covering the central 10° of retinal eccentricity was implemented, with a 4-2 threshold strategy, stimuli size Goldmann III, background luminance of 4 apostilb, maximum luminance of 1000 apostilb, and a 36-dB dynamic range.

### Structural Measurements

Advanced glaucoma module scans were obtained for all participants using the Heidelberg Spectralis HRA + OCT MultiColor device. Macular pigment is localized in the foveal and parafoveal regions, and therefore, these regions were prioritized for structural assessment. Ganglion cell complex (GCC) thickness and peripapillary retinal nerve fiber layer (RNFL) thickness was measured using the Spectralis automatic segmentation software for parafoveal and peripapillary retinal thickness analysis. For the parafoveal analysis, a 7-mm^2^ area scan centered over the fovea was obtained and GCC and RNFL thickness values were computed across the central 1°, 3°, and 6° of retinal eccentricity.

### Lens Autofluorescence

The scanning confocal biomicroscope Clearpath DS-120 (Freedom Meditech, Inc) was used to measure lens autofluorescence using previously described methods.^34^^,^^35^ Lens autofluorescence, which has been shown to correlate to Lens Opacities Classification System III grading,^36^ was measured as an objective indicator of crystalline lens status across participants to control for change in crystalline lens status over time, a factor known to impact MPOD measurement accuracy.^37^

### Statistical Methods

Sample size calculations for the ENIGMA study were conducted using GPower.^38^ The study was powered to detect a macular pigment response resulting from supplementation. The analysis was conducted with a power (1 – β) set at 0.95 and α = 0.05, 2-tailed, 2 groups, 4 repeated measures, a correlation among repeated measures of 0.5, and an effect size *f* set at 0.25. The estimated sample size required to detect a change in MPOD was 56 participants. Statistical analysis was performed using R software version 3.6.3 (R Foundation for Statistical Computing) in RStudio (RStudio Team 2020). The ezPerm function in ez package was used to run permutation tests on the mesopic CS with glare data.^39^ The ggplot package was used for plotting graphical representations.^40^

Baseline differences between intervention groups were assessed using independent samples *t* tests for normally distributed data and Mann–Whitney *U* tests used for nonnormally distributed data. A mixed repeated measures analysis of variance was used to assess change in MPOD and structural and functional measures over the study treatment period, with time as a within-subjects factor and intervention group as a between-subjects factor. All tests were 2-tailed, and a 5% level of significance was used throughout.

## Results

**Figure 1:**
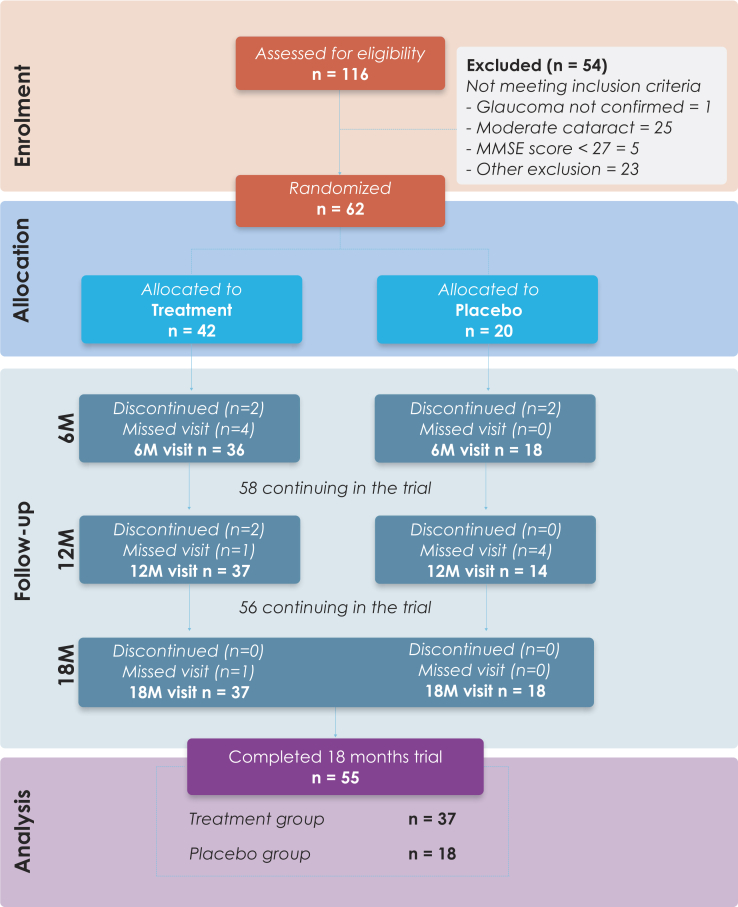
Consolidated Standards of Reporting Trials flow diagram illustrating participant progress through the European Nutrition in Glaucoma Management trial. M = months.

A total of 116 volunteer participants were screened for eligibility to participate in the ENIGMA study, of whom 62 were enrolled at baseline and were randomized to treatment or placebo intervention. The racial distribution was primarily White (n = 60), with just 1 Black participant and 1 Asian participant, reflecting the Irish population, which remains predominantly White (92.4%). A Consolidated Standards of Reporting Trials flow diagram illustrating the ENIGMA study design and participant progress through the enrolment, intervention allocation, follow-up, and data analysis phases of the trial is provided in Figure 1. Baseline demographic, MPOD, functional, and structural characteristics were statistically comparable between intervention groups (Table 1).

**Table 1:** Baseline Characteristics of Participants Enrolled in the European Nutrition in Glaucoma Management Study with Statistical Comparisons According to Treatment Allocation Group

Parameter (Baseline)	Active Group (n = 42)	Placebo Group (n = 20)	*P* Value∗
Age (yrs)†	66.23 ± 9.25	62.89 ± 12.53	0.53
Sex			0.27
Female	14	10	
Male	28	10	
BMI (kg/m^2^)‡	28.05 ± 5.55	26.64 ± 4.57	0.35
Hip-to-waist ratio‡	1.08 ± 0.08	1.1 ± 0.09	0.47
MMSE score‡	29.24 ± 1.12	29.41 ± 0.94	0.58
Education (yrs)‡	16.35 ± 4.63	16 ± 3.86	0.79
Smoking			0.50
Never	22	8	
Former	14	10	
Current	5	2	
Lutein, zeaxanthin, and meso-zeaxanthin dietary intake score‡	22.84 ± 12.72	28.78 ± 15.84	0.13
MPOD volume†	5316.03 ± 3066.69	4870.47 ± 2204.1	0.95
Visual acuity (VAR)‡	96.12 ± 7.6	94.28 ± 9.08	0.42
Microperimetry average threshold (dB)†	22.71 ± 4.99	24.21 ± 4.25	0.32
HVF 24-2 MD (dB)‡	–10.62 ± 6.57	-8.01 ± 7.19	0.24
GAL-9 questionnaire score†	15.46 ± 6.84	15.35 ± 8.31	0.55
Photopic contrast sensitivity with glare (logCS), cpd†			
1.5	1.2 ± 0.35	1.19 ± 0.33	0.75
3	1.47 ± 0.33	1.44 ± 0.42	0.83
6	1.5 ± 0.42	1.34 ± 0.5	0.17
12	1.0 ± 0.4	1.02 ± 0.47	0.54
18	0.53 ± 0.35	0.58 ± 0.37	0.65
Mesopic contrast sensitivity with glare (logCS), cpd†			
1.5	0.8 ± 0.36	0.99 ± 0.39	0.90
3	0.96 ± 0.43	1.14 ± 0.44	0.22
6	0.86 ± 0.43	0.9 ± 0.41	0.69
12	0.51 ± 0.18	0.58 ± 0.21	0.15
18	0.32 ± 0.1	0.35 ± 0.12	0.13
Lens autofluorescence‡	0.197 ± 0.05	0.17 ± 0.04	0.06
Macular RNFL thickness (μm), degrees†			
3	19.67 ± 2.66	19.18 ± 2.35	0.43
6	25.41 ± 5.49	25.36 ± 5.52	0.82
GCC thickness (μm), degrees‡			
3	87.7 ± 18.15	84.44 ± 16.65	0.53
6	79.36 ± 16.06	78.14 ± 12	0.80

BMI = body mass index; cpd = cycles per degree; GAL-9 = Glaucoma Activities Limitation 9 questionnaire; GCC = ganglion cell complex; HVF = Humphrey visual field; logCS = logarithm of contrast sensitivity units; MD = mean deviation; MMSE = Mini-Mental State Examination; MPOD = macular pigment optical density; RNFL = retinal nerve fiber layer; VAR = visual acuity rating (100 / 50 × logarithm of the minimum angle of resolution; a score of 100 corresponds with 20/20).

Data are presented as mean ± standard deviation, unless otherwise indicated.

∗ Significance set at *P* < 0.05.

† Independent-samples Mann–Whitney *U* test used for nonnormally distributed data.

‡ Independent samples *t* test used for normally distributed data. Chi square analysis used for categoric data.

### Compliance

A high degree of compliance to study supplement use was noted among participants in both groups (>80% tablets consumed by 54 of the 55 participants who completed the trial). One active treatment group participant was deemed noncompliant to study supplement use (<10% use despite repeated education on the potential benefits of good compliance) measured by tablet counting. This individual did not experience any change in MPOD, but is included in the intention-to-treat analyses presented herein.

### Macular Pigment Optical Density Response to Supplementation

**Figure 2:**
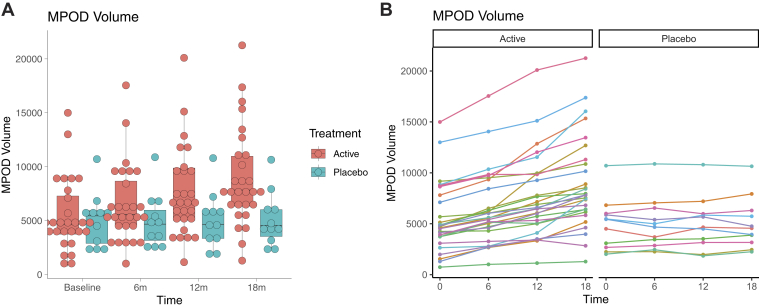
**A**, Boxplot of macular pigment optical density (MPOD) volume illustrating MPOD values for the carotenoid treatment group (red) and the placebo group (green) for the baseline period (0m), 6 months (6m), 12 months (12m), and 18 months. Dots represent individual participant values at each time point. **B**, Line graph illustrating individual MPOD change for each participant in the active treatment group (left panel) and placebo treatment group (right panel).

A 2 (treatment group: active carotenoids and placebo) by 4 (time: baseline [0 months], 6 months, 12 months, and 18 months) mixed repeated-measures analysis of variance revealed a statistically significant increase in MPOD volume (normalized square root MPOD volume) during the study period (significant time effect: *F*(3,111) = 89.31, MSE = 1656.9; *P* < 0.01) and a statistically significant interaction of time and intervention group (*F*(3,111) = 31.71, MSE = 588.4; *P* < 0.01). No significant difference in MPOD was observed between groups (nonsignificant group effect: *F*(1,37) = 2.64, MSE = 4658; *P* = 0.11). The change in MPOD volume over time in the treatment versus placebo group participants is illustrated as a boxplot in Figure 2, with participant MPOD values plotted as individual dots at each time point to demonstrate the general upward trend in MPOD for all compliant participants in the treatment group (note the 1 noncompliant participant whose MPOD remained stable throughout) and relative stability of MPOD among participants in the placebo group. The detailed statistical analysis of the change in MPOD is presented in Table 2.

**Table 2:** Mixed Repeated Measures Analysis of Variance of Change in Macular Pigment Optical Density from Baseline to 18 Months by Intervention Group

Macular Pigment Optical Density	Group 1 - Active				Group 2 - Placebo				Time Effect		Time × Group Interaction	
	Baseline	6 Months	12 Months	18 Months	Baseline	6 Months	12 Months	18 Months	*F*	*P* Value∗	*F*	*P* Value∗
0.23°	0.46 ± 0.2	0.51 ± 0.19	0.56 ± 0.20	0.63 ± 0.23	0.47 ± 0.20	0.47 ± 0.17	0.48 ± 0.19	0.47 ± 0.17	31.28	**<0.01**	12.94	**<0.01**
0.51°	0.40 ± 0.18	0.45 ± 0.19	0.50 ± 0.19	0.58 ± 0.22	0.41 ± 0.17	0.41 ± 0.17	0.42 ± 0.17	0.43 ± 0.16	48.14	**<0.01**	14.06	**<0.01**
0.74°	0.39 ± 0.19	0.44 ± 0.20	0.50 ± 0.20	0.56 ± 0.22	0.40 ± 0.18	0.40 ± 0.18	0.40 ± 0.18	0.40 ± 0.18	47.06	**<0.01**	19.59	**<0.01**
1.02°	0.34 ± 0.18	0.39 ± 0.18	0.44 ± 0.20	0.50 ± 0.21	0.34 ± 0.16	0.33 ± 0.17	0.34 ± 0.17	0.35 ± 0.16	49.12	**<0.01**	15.96	**<0.01**
Volume	5316.03 ± 3066.69	6627.87 ± 3630.9	7450.63 ± 3879.75	8530.43 ± 4370.36	4870.47 ± 2204.11	4742.61 ± 2276.77	5034.14 ± 2444.81	4847.59 ± 2343.98	89.31	**<0.01**	31.71	**<0.01**

Data are presented as mean ± standard deviation, unless otherwise indicated. Macular pigment optical density measurements were normalized for analysis purposes as a square root transformation. Number of participants who completed all 4 study visits included in the analysis of variance, n = 39 (treatment group, n = 28; placebo group, n = 11). Boldface indicates statistically significant *P* values.

∗ Significance set at *P* < 0.05.

**Figure 3:**
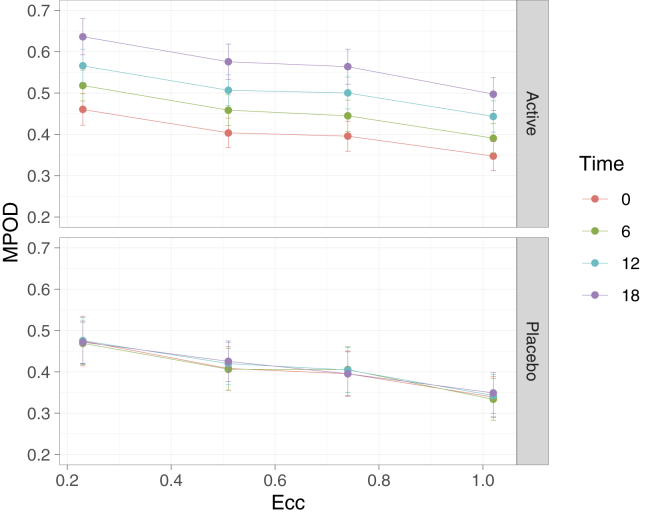
Graph showing change in macular pigment optical density (MPOD) across retinal eccentricity (Ecc) in the carotenoid treatment group (upper) and placebo group (lower) over time. Error bars represent standard error of mean.

Post hoc *t* tests revealed a significant difference in MPOD volume at each period for the treatment group, with MPOD increasing significantly at each 6-month visit (*P* < 0.01 for all), whereas no significant difference was found within the placebo group over time (*P* > 0.05 for all). Macular pigment optical density (overall volume and each retinal eccentricity) was statistically significantly higher in the treated group relative to the placebo group at 18 months (*P* < 0.01 for all), but not at other time points (*P* > 0.05 for all). The change in mean MPOD in the active treatment group over the 18-month supplementation period was consistent across the central 1.02° of retinal eccentricity, with average increases ranging from 0.16 to 0.18 optical density units across each retinal eccentricity (Fig 3).

**Figure 4:**
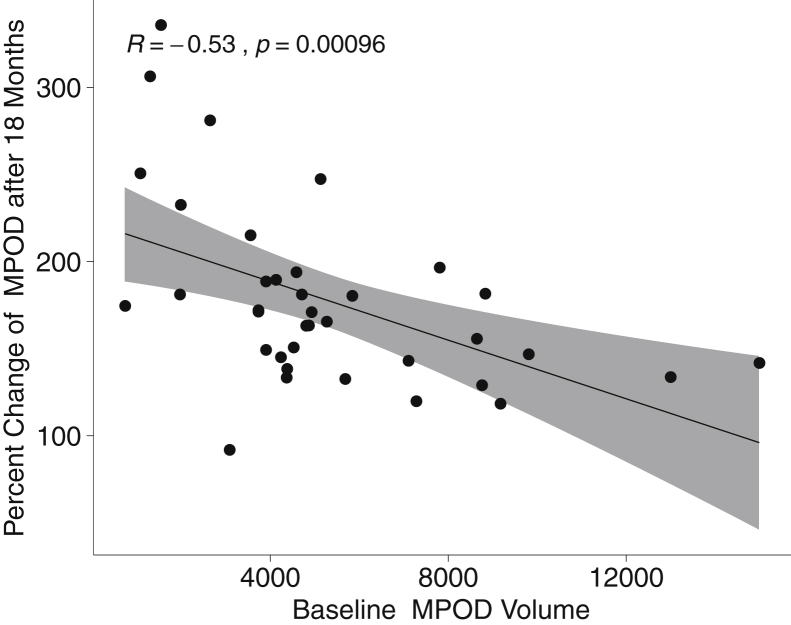
Scatterplot showing the percent change of macular pigment optical density (MPOD) volume after 18 months of carotenoid supplementation (treatment group) as a function of baseline MPOD volume.

In the carotenoid treatment group, an inverse and statistically significant relationship was observed between baseline MPOD volume and percentage change in MPOD volume over the 18-month supplementation period. Individuals with the lowest baseline MPOD exhibited a greater percentage improvement relative to baseline values compared with those with higher MPOD at baseline, indicating greater relative benefit among those with low MPOD before supplementation (Fig 4). Pearson’s correlation analysis revealed no significant association between baseline Humphrey visual field 24-2 mean deviation (an indicator of glaucoma severity) and change in MPOD over 18 months (*r*^2^ = 0.04; *P* = 0.27).

### Structure and Function Response

**Figure 5:**
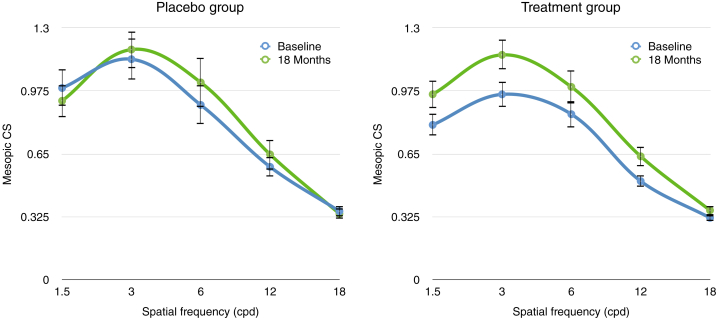
Graph showing the change in mesopic contrast sensitivity (CS) under glare conditions during an 18-month period of supplementation with macular carotenoids (lutein 10 mg, zeaxanthin 2 mg, and meso-zeaxanthin 10 mg) or placebo. Data are presented as mean CS at each spatial frequency. Error bars represent standard error of the mean. cpd = cycles per degree.

Preliminary analysis of the mesopic and photopic CS under glare data revealed significant improvements at 1.5 cpd, 3 cpd, 6 cpd, and 12 cpd from baseline to 18 months under mesopic conditions (*P* < 0.05 for all; Fig 5), but not photopic conditions (*P* > 0.05 for all). Floor effects were noted at high spatial frequencies (12 cpd and 18 cpd), however, because participants found higher-frequency stimuli difficult to resolve under glare conditions. Therefore, detailed analyses are confined to 1.5 cpd, 3 cpd, and 6 cpd.

To investigate the impact of treatment, time, and spatial frequency on mesopic contrast sensitivity under glare conditions, a 2 (treatment group: active carotenoids and placebo) by 2 (time: baseline 0 months and 18 months) by 3 (spatial frequency: 1.5 cpd, 3 cpd, and 6 cpd) mixed repeated-measures analysis of variance was conducted. The analysis revealed a statistically significant interaction of time and treatment group (*F*(1,225) = 5.35, MSE = 0.39; *P* < 0.05), a statistically significant effect of time (*F*(1,225) = 18.82, MSE = 1.36; *P* < 0.01), and a statistically significant effect of spatial frequency (*F*(2, 225) = 13.51, MSE = 0.98, *P* < 0.01). No significant difference in mesopic CS under glare was observed between groups (nonsignificant group effect: *F*(1,45) = 0.36, MSE = 0.28; *P* = 0.55) or any other interaction, time by frequency (*F*(2,225) = 0.28, MSE = 0.02; *P* = 0.76), treatment group by frequency (*F*(2,225) = 0.23, MSE = 0.016; *P* = 0.80), or treatment group by frequency by time (*F*(2,225) = 0.49, MSE = 0.04; *P* = 0.61). Post hoc permutation tests revealed a statistically significant interaction of time and treatment group in mesopic CS under glare conditions at 3 cpd (Table 3; Fig 5). No change was noted in the placebo group.

**Table 3:** Mixed Repeated Measures Analysis of Variance of Structural and Functional Measures from Baseline to 18 Months by Treatment Group

Variable	*Group 1 - Active*		*Group 2 - Placebo*		P *Value*∗		
	Baseline	18 Months	Baseline	18 Months	Time × Group Interaction	Group Effect	Time Effect
Mesopic CSg (cpd)							
1.5	0.78 ±0.35	0.96 ± 0.42	0.98 ± 0.41	0.92 ± 0.34	0.24	0.427	**<0.05**
3.0	0.96 ±0.44	1.16 ± 0.46	1.13 ± 0.46	1.17 ± 0.38	**< 0.05**	0.479	0.06
6.0	0.83 ± 0.43	1.02± 0.50	0.91 ± 0.42	1.01 ± 0.46	0.52	0.784	**<0.01**
Photopic CSg (cpd)							
1.5	1.2 ± 0.36	1.26 ± 0.38	1.19 ± 0.35	1.25 ± 0.28	0.924	0.906	**<0.05**
3.0	1.46 ± 0.37	1.52 ± 0.45	1.45 ± 0.44	1.46 ± 0.35	0.495	0.805	0.251
6.0	1.48 ± 0.46	1.42 ± 0.51	1.35 ± 0.53	1.26 ± 0.56	0.757	0.345	0.146
Microperimetry (dB)	22.7 ± 5.4	22.4 ± 6.6	24.5 ± 4.2	24.3 ± 4.1	0.52	0.28	**<0.01**
GCC thickness (μm), degrees							
1	48.0 ±10.9	49.7 ± 11.8	45.28 ± 9.7	43.8 ± 6.8	0.317	0.156	0.564
3	87.34 ± 18.38	86.1 ± 18.3	85.2 ±16.09	83.1 ± 13.4	0.561	0.643	**<0.05**
6	79.68 ±16.6	76.9 ± 13.3	78.1 ± 12.0	75.5 ± 8.59	0.973	0.716	**<0.05**
RNFL thickness (μm), degrees							
1	12.4 ± 2.2	13.1 ± 2.9	11.9 ± 3.0	12.8 ± 4.9	0.841	0.625	0.136
3	19.4 ± 2.7	19.6 ± 2.4	19.2 ± 2.6	19.4 ± 3.5	0.956	0.758	0.391
6	25.6 ± 5.7	25.9 ± 6.8	25.6 ± 5.6	23.9 ± 3.6	**< 0.05**	0.563	0.375
GAL-9 score	15.53 ± 6.758	15.125 ± 5.873	13.6 ± 5.9	12.8 ± 4.36	0.803	0.219	0.471
VAR	95.89 ± 7.8	96.25 ± 8.83	94.9 ± 9.27	94.4 ± 9.8	0.818	0.273	0.484

cpd = cycles per degree; CSg = contrast sensitivity under glare; dB = decibel; GAL-9 = Glaucoma Activity Limitation 9 questionnaire; GCC = ganglion cell complex; RNFL = retinal nerve fiber layer; VAR = visual acuity rating (100 / 50 × logarithm of the minimum angle of resolution; a score of 100 corresponds with 20/20).

Data are presented as mean ± standard deviation, unless otherwise indicated. Number of participants who completed all 4 study visits included in the analysis of variance, n = 47 (treatment group, n = 32; placebo group, n = 15). Microperimetry reported as the average threshold in decibels within the central 10°. Boldface indicates statistically significant *P* values.

∗ Significance set at *P* < 0.05.

Although some additional statistically significant time effects (GCC) and a significant time by group interaction (RNFL) were observed, no clinically meaningful structural or functional improvements were noted during the study period for VA, photopic CS under glare, microperimetry, or Glaucoma Activity Limitation 9 questionnaire score (*P* > 0.05 for all). Similarly, no significant changes in structural parameters including RNFL and GCC were observed in either intervention group (Table 3). Lens autofluorescence and dietary carotenoid intake remained unchanged in both groups throughout the study duration, and no significant differences were observed between baseline and 18 months in either group (*P* > 0.05 for all).

### Adverse Events

A small number of adverse events were reported during the course of the study, including mild nausea (n = 3; all from the treatment group), stomach upset (n = 3; n = 2 from the treatment group, n = 1 from the placebo group), and skin rash (n = 1 from the placebo group). The proportion of participants experiencing any adverse event was statistically similar between interventions: 5 of 42 participants (12%) from the treatment group and 2 of 20 participants (10%) from the control group. All adverse events were reported to resolve quickly and did not result in any participant withdrawal from the study. No serious adverse event relating to the study intervention was reported in either intervention group during the course of the study.

## Discussion

The ENIGMA trial provides novel insights into the effect of macular carotenoid supplementation on MP levels in glaucomatous eyes. The primary intention-to-treat analyses demonstrated a beneficial effect on MPOD of supplementation with 10 mg lutein, 2 mg zeaxanthin, and 10 mg meso-zeaxanthin. Specifically, a 60% mean increase in MPOD volume was observed over the 18-month trial duration among those randomized to receive the macular carotenoid supplement, whereas MPOD remained relatively unchanged among those assigned to placebo. A number of important observations can be made in relationship to the pattern of MPOD response detected over the course of the trial. The highest average rate of change was observed in the first 6 months (25% increase), but significant increases were observed at each time point thereafter, such that the benefit of supplementation was sustained throughout the 18-month treatment period. It is not possible, at this point, to determine the time course over which increases in MPOD might plateau if supplementation were continued indefinitely. The highest MPOD volume recorded in this study by any participant was 21 260, which represented a 42% increase from baseline levels at 14 991. The lowest recorded overall MPOD volume was approximately 30 times lower compared with this peak volume. Significant scope seems to exist for continued increase in MPOD, therefore, particularly among those with low baseline MPOD levels.

Interestingly, the relative MPOD enhancement was greatest amongst those with lowest baseline values, with some participants exhibiting more than 3-fold increases with respect to the baseline level. However, every compliant participant seemed to benefit in terms of increased MPOD, even those with the highest baseline levels. In contrast, nonresponders to supplementation have been identified in previous trials, including studies involving carotenoid supplementation among patients with AMD.^41^^,^^42^ Although the cause of variation in tissue response to carotenoid supplementation is poorly understood, use of a high-dose supplement (in this study, 22 mg total carotenoid dose per daily capsule) containing all 3 macular carotenoids seems to be associated with an enhanced MP response.^27^^,^^43^^,^^44^ That the increases observed were approximately equivalent at each eccentricity measured within the central 1.02° represents another possible benefit of including all 3 macular carotenoids in the supplement formulation. However, the beneficial MPOD response observed herein seems generalizable to other supplement formulations based on a recent supplementation study among participants with AMD. In this study, the AREDS2 supplement formulation, which contains 10 mg lutein and 2 mg zeaxanthin plus other vitamins and micronutrients, was compared with the same 22-mg formulation used herein. No difference was observed in MPOD response between the 2 different formulations over a 2-year supplementation period.^45^

These MPOD response findings are important in the context that MPOD seems to be lower among individuals with glaucoma. Although 1 study failed to demonstrate any relationship between glaucoma and MPOD volume (participants had earlier stage glaucoma and more than 30% of trial participants with glaucoma were already supplementing with carotenoids, which represents a major confounder),^46^ a number of studies have shown that MPOD is depleted in patients with glaucoma compared with age-matched control participants,^24^^,^^25^ whereas those with GCC loss involving the foveal zone seem to have lower MPOD compared with those without foveal GCC involvement.^20^ Interestingly, low macular pigment also recently was identified as a risk factor for primary OAG among older women in the Carotenoids in Age-Related Eye Disease Study (CAREDS).^47^ In this longitudinal cohort study, MPOD levels initially were captured in the CAREDS baseline study (2001–2004) and again in the CAREDS 2 follow-up study (2016–2019). Specifically, among 630 participants eligible for inclusion in the CAREDS glaucoma study, women in the lowest quartile of baseline MPOD were significantly more likely to have manifest glaucoma than those in other quartiles (*P* = 0.04). The inverse association between MPOD and glaucoma was strongest among eyes exhibiting stable MPOD at both visits (*P* < 0.01). Macular pigment optical density depletion conceivably may result from several causal mechanisms applicable to glaucoma. These include: (1) inadequate dietary intake or overuse of available carotenoid stores to combat oxidative stress and inflammation,^23^^,^^48^ (2) undersupply as a consequence of vascular dysregulation,^49^ and (2) inadequate structural housing to support binding of these antioxidant nutrients in inner retinal layers damaged by glaucoma.^23^ The positive MPOD response observed herein is important, therefore, because it demonstrates that MPOD can be augmented readily through supplementation in patients with OAG, regardless of both disease severity (as indicated by mean deviation values on Humphrey visual fields) and of any disease-specific mechanisms that may be involved in determining individual baseline levels. Dual-wavelength autofluorescence seems to provide a reliable method for establishing MPOD response to treatment. However, the safety of using this photobleaching method for long-term MPOD monitoring in diseased eyes does need to be explored.

The observation that MPOD responds to supplementation in glaucoma is of specific interest because MP exhibits specific biological qualities that may confer neuroprotective benefits in the glaucomatous eye.^50^, ^51^, ^52^, ^53^ Neuroprotection is a potentially important therapeutic area designed to target molecular pathways of RGC damage in glaucoma treatment.^54^ Hence, compounds such as the macular carotenoids that prevent or slow down apoptosis-inducing pathways such as ischemia, oxidative stress, inflammation, and mitochondrial dysfunction may confer a neuroprotective effect.^55^ Oxidative stress and chronic inflammation are key pathways of tissue damage commonly involved in degenerative ophthalmic conditions, including glaucoma. Oxidative DNA damage is elevated in glaucoma,^56^^,^^57^ and reactive oxygen species cytotoxicity is directly involved in RGC death.^58^, ^59^, ^60^ Inflammation, which is the body’s response to ischemic injury, also plays an important role in glaucoma pathogenesis and may link increased intraocular pressure and ischemia directly with RGC loss^61^ and may induce proapoptotic reactions in the RGCs through the release of proinflammatory cytokines.^62^ Optically, MP acts as a prereceptoral filter that limits retinal exposure to high-energy short-wavelength blue light, and thereby has the capacity to limit light-mediated oxidative damage at the macula.^63^ Additionally, as potent antioxidant and anti-inflammatory nutrients,^53^ lutein, zeaxanthin, and meso-zeaxanthin may support RGCs and may confer protection in glaucomatous eyes by inhibiting reactive oxygen species and preventing the pathophysiologic cascades of oxidative stress and inflammation.^64^

The functional improvement in glare-affected CS under mesopic conditions observed herein represents another potentially important finding, particularly given that the ENIGMA trial was not powered to detect functional or structural changes in response to supplementation. Although the disease can be asymptomatic in the early stages, problems relating to glare disability and dark adaptation are reported commonly by individuals with glaucoma, even in those with mild visual field loss.^30^^,^^65^ Despite the negative quality-of-life impact associated with glare disability in patients with glaucoma,^30^ these symptoms are not typically addressed with current therapy. Lower MP levels previously were shown to be associated with glare-affected visual function in glaucoma.^33^ The effect of lutein, zeaxanthin, and meso-zeaxanthin supplementation on the symptoms of glare in glaucoma has not been explored previously, but it is widely recognized that higher MPOD is associated with better visual performance.^27^^,^^66^^,^^67^ Prereceptoral absorption of short-wavelength blue light by MP is known to have a direct influence on visual performance, whereby macular pigment has the capacity to optimize and refine the visual signal to be delivered along the visual pathway. These optical effects may explain the improvements in glare-related performance observed herein,^51^ which seem to mirror glare-related performance enhancements reported elsewhere as a beneficial outcome of lutein, zeaxanthin, and meso-zeaxanthin supplementation in healthy individuals^68^^,^^69^ and in those with AMD.^45^

The structural and functional outcomes reported herein need to be interpreted with caution, however. First, the sample size was too small to accept or reject the null hypothesis reliably that MP augmentation might yield structural or functional benefits. Although the improved glare performance observed herein may reflect increased prereceptoral short-wavelength light absorption, it is important to note that the limited 18-month duration of the ENIGMA trial represents a significant limitation with respect to determining any structural or functional benefits that might accrue as a consequence of the neuroprotective influence of MP over time. Nutritional supplementation trials for glaucoma (which attract less funding compared with drug trials) have attracted criticism in relationship to the characteristically small sample sizes and short treatment duration used.^70^ This is particularly problematic in glaucoma, which is a chronic and slowly progressive disease in which functional performance exhibits a high degree of measurement variability.^71^ In visual field assessment, for example, a minimum 2-year follow-up period has been suggested to detect functional loss reliably,^72^^,^^73^ whereas clinical trials designed to investigate the neuroprotective benefits of treatments such as brimonidine have illustrated functional benefits only after at least 2 years of follow-up.^74^

In conclusion, the ENIGMA trial provides clear evidence that MPOD can be augmented in individuals with OAG. Therefore, macular carotenoid supplementation may prove useful as an adjunct therapy to optimize visual function and to preserve macular health in glaucoma. A supplement formulation containing 10 mg lutein, 2 mg zeaxanthin, and 10 mg meso-zeaxanthin can generate a sizeable and continuous increase in overall MPOD throughout an 18-month treatment window independent of baseline MPOD levels, so the potential benefits are not reserved for those with low MP. This is important in the context of recent evidence linking low MP to OAG.^20^^,^^24^^,^^25^^,^^47^ Although structural integrity and functional performance remained relatively unchanged, the improvement in glare-affected mesopic CS is noteworthy, particularly in the context of the glare-related symptoms known to affect people with glaucoma. These findings support the observation in a recent review that a strong rationale exists for continuing to explore the importance of MP in OAG.^55^ However, many research questions remain unanswered, including the need for additional confirmatory evidence that MP deficits are associated with OAG, particularly when measured by autofluorescence. Future intervention trials certainly should prioritize an appropriately powered study with a minimum 2- to 5-year treatment window to explore the potential long-term benefits of these potent antioxidant and anti-inflammatory nutrients for visual function and ocular health in glaucoma, with particular emphasis on glare-affected visual function.
